# Mechanism of action of over-expressing NY-ESO-1 in vesicular stomatitis virus and its combination therapy with NY-ESO-1 TCR-T

**DOI:** 10.3389/fimmu.2025.1617941

**Published:** 2025-08-12

**Authors:** Ting Tian, Liang Ma, Liying Mao, Xiangxiang Wang, Longxin Cheng, Qibin Ma, Rong Xu, Guoqing Zhou

**Affiliations:** ^1^ Research and Development Department, Joint Biosciences (SH) Ltd, Shanghai, China; ^2^ Scientific Advisory Board, Joint Biosciences (SH) Ltd, Shanghai, China

**Keywords:** vesicular stomatitis virus, NY-ESO-1, tumor, TCR-T cell, combination (combined) therapy

## Abstract

**Introduction:**

Vesicular stomatitis virus (VSV) is a promising oncolytic viral platform due to its short replication cycle, broad tissue tropism, low natural infection rate in humans, and a small genome that is easy to genetically manipulate. Leveraging these advantages, we developed an attenuated oncolytic VSV-based virus, OVV-01, encoding the tumor-associated antigen (TAA) NY-ESO-1.

**Methods:**

OVV-01 was constructed by inserting the NY-ESO-1 gene into a VSV backbone. *In vitro* cytotoxicity assays were performed across various tumor cell lines to evaluate its oncolytic activity. The expression and presentation of NY-ESO-1 on infected cells were assessed. *In vivo* studies using xenograft mouse models were conducted to examine tumor selectivity, T cell activation, and therapeutic efficacy, both alone and in combination with NY-ESO-1-specific TCR-engineered T cells.

**Results:**

OVV-01 efficiently infected and inhibited the growth of multiple tumor cell lines *in vitro*. The overexpressed NY-ESO-1 was presented on the tumor cell surface and recognized by NY-ESO-1-specific TCR-T cells, promoting targeted cytotoxicity. In vivo, OVV-01 selectively replicated in tumor tissues and induced stronger activation of hCD4⁺, hCD8⁺, and NY-ESO-1-specific TCR-T cells compared to the control virus OVV-00. Combination therapy with OVV-01 and TCR-T cells significantly enhanced tumor control compared to monotherapies.

**Discussion:**

Our findings demonstrate that OVV-01 not only possesses potent direct oncolytic activity but also enhances the efficacy of adoptive T cell therapy by improving antigen presentation and T cell activation. This dual mechanism provides a rationale for using OVV-01 in combination immunotherapy strategies targeting solid tumors.

## Introduction

Oncolytic virotherapy has emerged as a novel and promising strategy in cancer treatment, leveraging genetically modified viruses to selectively infect and lyse tumor cells while stimulating systemic antitumor immune responses. Over the past two decades, a variety of oncolytic viruses (OVs) have been explored as innovative forms of cancer immunotherapy. Viral platforms under development include adenovirus, herpes simplex virus (HSV), measles virus, coxsackievirus, poliovirus, reovirus, vaccinia virus, Newcastle disease virus, among others (1). Several of these platforms have progressed into early- and late-stage clinical trials, and some have achieved regulatory approval in specific indications, such as Oncorine (2) (recombinant human adenovirus type 5), T-VEC (3) (HSV-1), and Delytact (4) (HSV-1). Different oncolytic viruses offer distinct advantages based on their replication kinetics, genome structure, immunogenicity, and ability to carry therapeutic transgenes. Adenoviruses are valued for their broad host range and gene delivery capacity, while HSV-based viruses (e.g., T-VEC and Delytact) exploit the natural cytolytic properties of HSV and allow for large-scale genome editing (5, 6). Despite this diversity, optimizing viral vectors for efficient tumor targeting, immune activation, and large-scale production remains a challenge.

In this context, vesicular stomatitis virus (VSV) has emerged as a highly promising next-generation oncolytic platform due to several distinct advantages over more established vectors such as adenovirus and poxvirus. VSV possesses a small, negative-strand RNA genome that is easy to manipulate genetically and supports rapid replication independent of the host cell cycle (7–9). Its ability to replicate efficiently across a wide range of tumor cell lines enables high-titer production, facilitating large-scale manufacturing. Importantly, VSV replicates exclusively in the cytoplasm without integrating into host DNA, thus eliminating concerns regarding insertional mutagenesis (8, 10–13). Compared with other viral platforms, VSV also exhibits low pre-existing seroprevalence in humans and relatively low immunogenicity, reducing the risk of early neutralization by the host immune system (8, 14, 15).

Moreover, advances in genetic engineering have enabled attenuation of VSV’s natural neurovirulence, further improving the safety profile of recombinant VSV-based vectors (8, 16, 17). Moreover, advances in genetic engineering have enabled attenuation of VSV’s natural neurovirulence, further improving the safety profile of recombinant VSV-based vectors (18).

Vesicular stomatitis virus (VSV) belongs to the family *Rhabdoviridae* and the genus *Vesiculovirus*. It is an enveloped, single-stranded, negative-sense RNA virus. VSV is composed of five genes (N, P, M, G, and L), which encode the nucleocapsid protein, phosphoprotein, matrix protein, glycoprotein, and large protein, respectively (19, 20). The matrix protein is responsible for forming the viral core and anchoring the glycoprotein to the viral membrane, thereby forming glycoprotein trimers. The glycoprotein determines receptor recognition, cell entry, and viral fusion, making it the primary target of humoral immune responses. Viral RNA replication, dependent on RNA polymerase activity, occurs in the cytoplasm of target cells and is driven by a complex containing the nucleoprotein, viral polymerase, and phosphoprotein (21, 22).

In 2000, Stojdl et al. first demonstrated the potential of VSV as an oncolytic virus, showing that VSV significantly inhibited melanoma growth in nude mice while having minimal effects on normal tissue cells (16). A key advantage of VSV is its ability to selectively replicate in tumor cells, which forms the foundation of its oncolytic activity (23). The selective replication of VSV in tumor cells is primarily attributed to impairments in the type I interferon (IFN-I) signaling pathway, which compromise the antiviral response in these cells (16, 23, 24). VSV achieves its oncolytic effects by activating multiple caspase-dependent apoptotic cascades, including high efficacy against tumor cells carrying activated oncogenes (e.g., Ras and Myc) or inactivated tumor suppressor pathways (e.g., p53) (10).

To date, the clinical safety of VSV as the backbone vector has been extensively studied. From 2010 to 2024, a total of 62 clinical studies involving the VSV backbone have been conducted in cancer therapy as well as in vaccine development for Ebola virus, COVID-19, and HIV. 23 VSV-based oncolytic viruses have been evaluated in clinical trials for cancer treatment (data sourced from https://classic.clinicaltrials.gov/). For example, investigators at the Mayo Clinic has developed a genetically modified vesicular stomatitis virus, VSV-IFNβ-NIS, which has completed a Phase I clinical trial in patients with relapsed or refractory T-cell lymphoma (TCL), showing a good safety profile and promising efficacy. This virus has also shown promising results in early-phase I/II trials in combination with immune checkpoint inhibitors (25).

New York Esophageal Squamous Cell Carcinoma Antigen 1 (NY-ESO-1) is a tumor-associated antigen (TAA) that has been studied for over 20 years. Due to its restricted expression in germ cells and a wide range of human tumors, and its absence in normal tissues, NY-ESO-1 has emerged as one of the most promising antigens for cancer-specific immune recognition (26). It is expressed in approximately one-quarter to one-third of tumor cells include lung cancers, melanomas, ovarian cancers, esophageal cancers, bladder cancers, and prostate cancers, and its expression is associated with high-grade tumors. NY-ESO-1 also exhibits spontaneous immunogenicity in some patients, eliciting CD8+ T cell and CD4+ T cell responses, which are linked to the presence of NY-ESO-1 antibodies in the serum (27). Given its tumor-specific expression and strong immunogenic potential to induce both humoral and cellular immune responses, NY-ESO-1 has been explored as a target for modulating the tumor immune microenvironment (28, 29). Numerous clinical studies targeting NY-ESO-1 have been initiated (28, 30).

Wild-type VSV, upon entering host cells, can inhibit host mRNA nuclear export and disrupt host transcriptional activity, thereby suppressing the host’s antiviral response. The M protein plays a critical role in this process. VSV with an M51R mutation in the M protein has a reduced ability to inhibit host gene expression, allowing viral replication to occur exclusively in tumor cells and tissues and enhancing the virus’s safety profile. Common strategies for engineering oncolytic viruses to carry exogenous genes often focus on expressing immune-activating factors or immune checkpoint inhibitors such as GM-CSF, IFNβ, IL12, IL15, or PD1/PDL1. In contrast, strategies involving antigen expression are less frequently reported. Here, we introduce the OVV-Drive-IO™ approach, which involves using an oncolytic virus to express the highly immunogenic endogenous antigen NY-ESO-1, thereby constructing an oncolytic virus vaccine (OVV) to enhance anti-tumor immune activation.

Adoptive T cell therapies, including chimeric antigen receptor (CAR)-T cells and T cell receptor (TCR)-engineered T cells, have emerged as powerful immunotherapeutic strategies in cancer treatment. However, their clinical efficacy in solid tumors remains limited due to multiple tumor immune evasion mechanisms (31). Tumor cells can downregulate major histocompatibility complex class I (MHC-I) molecules, tumor-associated antigens (TAAs), and components of the antigen processing and presentation machinery, enabling them to escape T cell-mediated recognition and cytotoxicity (32, 33). Such immune escape not only limits the direct cytotoxic effects of adoptive T cell therapies but also contributes to tumor progression and treatment resistance.

Oncolytic virotherapy offers a promising strategy to overcome these limitations. Among various oncolytic viruses, vesicular stomatitis virus (VSV) is notable for its selective tumor cell infection, rapid replication, and low pre-existing immunity in humans. Beyond direct tumor lysis, oncolytic viruses infection induces immunogenic cell death and facilitates the release of TAAs, contributing to remodeling of the immunosuppressive tumor microenvironment (TME) (34, 35). This process can enhance T cell infiltration and activation within the tumor site, helping to overcome barriers associated with immune exclusion and T cell dysfunction.

Notably, recent studies have shown that engineered VSV vectors, such as VSVΔM51, can drive CD8^+^ T cell-mediated tumor regression not only through direct infection of tumor cells but also by targeting non-tumor stromal cells within the TME (36). This dual infection amplifies local inflammation and antigen presentation, thereby promoting improved T cell recruitment and activation. These findings highlight VSV’s dual role as both an oncolytic agent and an immune modulator, supporting its use in combination with adoptive T cell therapies.

Building on this concept, our study employs a recombinant VSV-based oncolytic virus vaccine (OVV-01) engineered to overexpress NY-ESO-1, a well-characterized TAA. It marks cancer cells by inducing the expression of NY-ESO-1 in tumors that are limited in therapeutic targets (referred to as “Mark”), and it can be combined with NY-ESO-1-specific TCR-T cell therapy to maximize tumor cell killing (referred to as “Kill”). This approach significantly expands the range of treatable tumor types and achieves a synergistic effect, where the combined treatment could yield efficacies greater than the sum of its parts. We refer this combined therapeutic strategy as “Mark + Kill.”

In this study, Our results show that OVV-01 exhibits potent tumor cytotoxicity both *in vitro* and *in vivo*. Immunohistochemical analysis confirmed its tumor-selective targeting capability in tumor tissues. Moreover, OVV-01 effectively enhances immune responses and, when combined with TCR-T cell therapy, synergistically augments the antitumor efficacy of TCR-T cells.

## Materials and methods

### pJB-NY-ESO-1 plasmid construction

Based on the genomic sequence of the VSV serotype strain Mudd-Summer, the genes encoding the M, P, N, G, and L proteins were codon optimized. The optimized viral genome was obtained through gene synthesis, and multiple mutations were introduced. The plasmids were transfected into BSR-T7 cells to produce various recombinant VSV strains. By screening and comparing the oncolytic efficacy and safety of these recombinant VSV strains, an attenuated strain with both favorable safety and potent tumor-killing capabilities was selected as the VSV backbone. The corresponding backbone plasmid, designated as pJB-00, was linearized by double digestion with XhoI and NheI. Using the primers 5’- CCGCTCGAGATGCAGGCAGAAGGAAGA -3’ and 5’- CTAGCTAGCTCATCTTCTCTGTCCGCT -3’, the NY-ESO-1 gene was amplified from the pUC19-NY-ESO-1 plasmid (NY-ESO-1 synthesized by GenScript, Suzhou, China) with DNA polymerase (NEB). The first primer included an XhoI site and the first 18 nucleotides of the NY-ESO-1 coding region, while the second primer included an NheI site and the last 18 nucleotides of the NY-ESO-1 gene. The PCR product was digested with XhoI and NheI and then cloned into the XhoI- and NheI-digested pJB-00 vector. The resulting plasmid was named pJB-NY-ESO-1.

### Recovery OVV-00 and OVV-01

BSR-T7 cells were cultured in MEM medium supplemented with 10% fetal bovine serum (FBS, Gibco) (complete medium). When the cells reached approximately 80% confluence, they were trypsinized, counted, and seeded into 6-well plates. The cells were then infected with vaccinia virus VTF7–3 at an MOI of 1. After 4 hours, the plasmids pN, pP, pL, pG, and pJB-NY-ESO-1 were transfected into the cells. The plasmids and their amounts used were as follows: 3 µg of pJB-00 or pJB-NY-ESO-1, 1.5 µg of pP, 1.2 µg of pN, 0.3 µg of pL, and 0.2 µg of pG. Transfection was performed using Lipofectamine^®^ LTX & PLUS™ Reagent (Invitrogen). After 48 hours after transfection (incubated in a 5% CO2, 37°C incubator), the cells and supernatant were collected, subjected to three freeze-thaw cycles, and centrifuged at 1200 rpm for 5 minutes. The supernatant was then filtered through a 0.22 µm filter to remove vaccinia virus particles. 1mL of the viral solution was added to a 6-well plate seeded with 1.5×10^6^ HEK 293 cells (ATCC), and the culture medium was replenished. The plate was incubated in a 5% CO2, 37°C incubator for approximately 48 hours. The cells and supernatant were collected, subjected to freeze-thaw cycles, filtered through a 0.22 µm filter, and stored at -80°C. The viruses were plaque-purified, and stable virus strains OVV-00 (derived from pJB-00 transfection) and OVV-01 (derived from pJB-NY-ESO-1 transfection) were obtained. After expansion and purification, the viruses were stored at -80°C, and their titers were determined using the TCID50 method (37, 38).

### *In vitro* cytotoxicity assay of OVV-00 and OVV-01 against tumor cells

The mouse breast tumor cell line 4T1 and the human cervical cancer cell line HeLa were obtained from ATCC, the mouse colon cancer cell line MC-38 and the mouse embryonic fibroblast (MEF) cell line were obtained from Nanjing Cobioer Biosciences CO., LTD (Nanjing, China). The mouse lung cancer cell line LLC was obtained from Cyagen Biosciences (Shuzhou, China). The colon cancer cell lines HCT116, HT-29, SW480, SW620, Colo205, and T84, as well as the lung cancer cell lines NCI-H446, A549, NCI-H460, HCC827, NCI-H596, and NCI-H1975, were obtained from PharmaLegacy Laboratories (Shanghai) Co., Ltd. 4T1 cells were cultured in RPMI-1640 (Gibco) supplemented with 10% FBS (Gibco). MC-38, MEF and LLC cells were cultured in DMEM (Gibco) supplemented with 10% FBS (Gibco). Hela cells were cultured in MEM (Gibco) supplemented with 10% FBS. HCT116 and HT-29 cells were cultured in McCoy’s 5a medium supplemented with 10% FBS. T84 cells were cultured in DMEM supplemented with 10% FBS. A549 cells were cultured in F-12K medium supplemented with 10% FBS. Colo205, SW480, SW620, HCC827, NCI-H1975, NCI-H446, NCI-H460, and NCI-H596 cells were cultured in RPMI-1640 medium supplemented with 10% FBS.

MTT method: After trypsinized, the MEF, LLC, MC-38, 4T1, and HeLa cells were counted and adjusted to a concentration of 1×10^5^ cells/mL. Then, 100 µL of the cell suspension was seeded into a 96-well plate and incubated in a humidified incubator at 37°C with 5% CO_2_ for 18–24 hours. OVV-00 and OVV-01 viruses were diluted in DMEM, RPMI 1640, or MEM medium supplemented with 2% FBS to a concentration of 1×10^4^ PFU/mL, respectively. After removing the culture supernatant, the viruses were added to the corresponding wells and incubated in the incubator for 24 hours. The virus inoculum was aspirated, and 100 µL of fresh culture medium was added to each well. Then, 10 µL of MTT solution (5 mg/mL) was added to each well, mixed, and incubated in the 37°C CO_2_ incubator for 4 hours. Afterward, the medium was removed, and 100 µL/well of SDS-HCl solution was added. The plate was mixed thoroughly and incubated at 37°C for 4 hours. The absorbance was measured at 570 nm using a microplate reader.

CTG method: Colo205, HCT-116, SW480, SW620, T84, HT-29, A549, HCC827, NCI-H1975, NCI-H446, NCI-H460, and NCI-H596 cells were seeded at 1.0×10^4^ cells per well in a 96-well plate with 90 µL per well. OVV-01 virus was diluted to 1.0×10^7^ PFU/mL and then serially diluted in 3-fold steps to create 9 different concentrations. 10 µL from each dilution gradient was added to the corresponding wells, with 3 replicates for each gradient. The plate was incubated at 37°C with 5% CO_2_ for 48 hours. After incubation, 100 µL of pre-warmed and room temperature equilibrated CTG (CellCounting-Lite^®^ 2.0 Luminescent Cell Viability Assay, Vazyme, Nanjing, China) solution was added to each well, and the plate was mixed for 2 minutes on a microplate shaker. After standing at room temperature for 10 minutes, the fluorescence signal was measured using a microplate reader (TECAN).

### Immunohistochemical Study of NY-ESO-1 expression in H22 tumor-bearing mice after single intravenous administration of OVV-01

The mouse liver cancer cell line H22 was purchased from Nanjing Cobioer Biosciences Co., Ltd., and cultured in RPMI 1640 medium supplemented with 10% FBS. Female Balb/c nude mice aged 6–8 weeks and weighing 18-20g, were obtained from Zhejiang Vital River Laboratory Animal Technology Co., Ltd. The mice were placed in controlled environments (12-h light/dark cycle; 20-26°C; 40-70% humidity) and had free access to bacteria-free water and food.

H22 cells were subcutaneously injected into mice at a dose of 2 × 10^5^ cells per mouse, with a volume of 100 μL per injection. When the tumor volume reached approximately 200 mm³, 7 mice were randomly assigned to 3 groups with 3 mice per treatment group and 1 mouse in control group.

Mice in the control group received an intravenous injection of 100 µL vehicle, while those in the treatment group were administered a single intravenous dose of 3×10^8^ PFU of OVV-01 per mouse. The tissues from the heart, lungs, liver, spleen, kidneys, uterus, brain, and tumor were collected at 0h (control group), 7h (group1), and 48h (group2) post the administration for histopathological analysis. Immunohistochemistry was performed to detect NY-ESO-1 protein expression using an anti-CTAG1B antibody (NY-ESO-1, Abcam) and fluorescence imaging scanner, 3DHISTECH; fully automated immunohistochemistry staining machine, Leica; Halo analysis software, Indica Lab.

At the end of the experiment, animals were euthanized using an overdose of CO_2_.

### Efficacy study of human PBMC-reconstituted CDX model in B-NDG HLA A2.1 mice

Female B-NDG HLA A2.1 mice and human PBMCs were provided by Biocytogen Jiangsu Gene Biotechnology Co., Ltd. The mice aged 6–8 weeks and weighing 16-24g, were placed in controlled environments (12-h light/dark cycle; 20-26°C; 40-70% humidity) and had free access to bacteria-free water and food. PBMCs were resuspended in serum-free RPMI 1640 medium and injected intravenously into B-NDG HLA A2.1 mice at a concentration of 5×10^6^ cells/0.2 mL per mouse. On the same day, HCT116 cells, resuspended in PBS, were injected subcutaneously into the right flank of the B-NDG HLA A2.1 mice (which had been injected with PBMCs) at a concentration of 5×10^6^ cells/0.1 mL per mouse. When the average tumor volume reached approximately 144 mm³, 27 mice were selected based on tumor volume and body weight, and randomly assigned to 3 groups with 9 mice per group. On Day 0, the OVV-00 and OVV-01 treatment was begun with 3×0^8^ PFU per mouse, administered intratumorally in 50 μL, while the vehicle control group received 50 μL of dilution medium intratumorally. Treatments were given every 2 days for a total of 3 times. On Day 7, Day 14, and Day 21, 3 mice from each group (9 mice total) were selected, blood was collected from the inner canthus for flow cytometry analysis, and tumor tissues were collected for TILs detection. The markers detected included: L/D (eBioscience™ Fixable Viability Dye eFluor™ 780), mCD45 (Biolegend, PerCP anti-mouse CD45), hCD45 (eBioscience Anti-Hu CD45, eFluor 506), hCD3 (eBioscience, Anti-Hu CD3 Super Bright 600), hCD4 (Biolegend, Alexa Fluor 488 anti-human CD4), hCD8 (Biolegend, APC anti-human CD8a), hFoxp3 (eBioscience, Anti-Hu Foxp3, PE/CyanineTM 7), and TCR (MBL, iTAgTM MHC Tetramer HLA-A* 02:01 NY-ESO-1 SLLMWITQC-PE). At the end of the experiment, animals were euthanized using an overdose of CO_2_.

### *In vitro* cytotoxicity assay of OVV-01 combined with NY-ESO-1-specific TCR-T cells

The A673, K562, K562-NY (NY-ESO-1 overexpressing), and Caski cells, as well as TCR-T cells (NY-ESO-1 TCR-T cells), used in the OVV-01 and TCR-T combination therapy study, were provided by TCRCure (Chongqing, China). A673 cells were cultured in DMEM (Gibco) supplemented with 10% FBS, K562 and K562-NY cells were cultured in IMEM (Gibco) supplemented with 10% FBS, and Caski cells were cultured in RPMI-1640 medium supplemented with 10% FBS. TCR-T cells were cultured in X-VIVO medium (Lonza) with the addition of 1000 IU/mL IL-2 (Bjshsw). All cells were maintained in a humidified incubator at 37°C with 5% CO_2_.

T-cell activation assay: OVV-01 oncolytic virus(MOI = 0, 2, 5)was first co-cultured with target cells A673, K562, and K562-NY (NY-ESO-1 overexpressing) for 24 h. Then T cells and target cells were co-cultured at different E:T ratios in the presence of the protein transport inhibitor Brefeldin A Solution (BFA, 1000X, ThermoFisher). After 5 hours, IFNγ and CD107a were detected.

While during *in vitro* cytotoxicity assay of OVV-01 and T cells against target cells: OVV-01 oncolytic virus(MOI = 0, 2, 5)was first co-cultured with target cells A673, K562, and K562-NY (NY-ESO-1 overexpressing) for 24 h. Target cells were labeled with CellTrace Violet (CTV) and then co-cultured with T cells at different E:T ratios. After 48h, cytotoxicity was analyzed by flow cytometry.

Flow cytometry antibodies: NY-ESO-1 monoclonal antibody (E978, Invitrogen), APC anti-human HLA-A2 antibody (Biolegend), PE anti-human IFN-γ antibody (Biolegend), PC5.5 anti-human CD107a (LAMP-1) antibody (Biolegend), 7-AAD Viability Staining Solution (Biolegend). Protein transport inhibitor: Brefeldin A Solution (1000X, ThermoFisher). Cell proliferation marker: CellTrace™ Violet Cell Proliferation Kit (CTV, ThermoFisher). Flow cytometer: Beckman CytoFLEX.

### Tumor growth inhibition experiment in H22 tumor-bearing mice treated with OVV-00 and OVV-01

The mouse liver cancer cell line H22 was purchased from Nanjing Cobioer Biosciences Co., Ltd., and cultured in RPMI 1640 medium supplemented with 10% FBS. Female Balb/c nude mice aged 6–8 weeks and weighing 18-20g, were obtained from Zhejiang Vital River Laboratory Animal Technology Co., Ltd. The mice were placed in controlled environments (12-h light/dark cycle; 20-26°C; 40-70% humidity) and had free access to bacteria-free water and food.

H22 cells were harvested and resuspended in RPMI-1640 basal medium and subcutaneously injected into mice at a dose of 2×10^5^ cells per mouse. When the tumor volume reached approximately 60 mm³, 24 mice were selected were randomly assigned to 3 groups with 6 mice per group. Mice in the control group received an intravenous injection of 100 µL vehicle, while those in the treatment group were administered intratumorally OVV-00 and OVV-01 at a dose of 3×10^6^ PFU per mouse, every 2 days for a total of 7 doses. Tumor volume was measured three times per week, and clinical symptoms were recorded daily.

At the end of the experiment, animals were euthanized using an overdose of CO_2_.

### Tumor growth inhibition experiment in tumor-bearing mice treated with OVV-01 and NY-ESO-1 TCR-T cells

Caski cells, as well as TCR-T cells (NY-ESO-1 TCR-T cells) were provided by TCRCure (Chongqing, China). Caski cells were cultured in RPMI-1640 medium supplemented with 10% FBS. TCR-T cells were cultured in X-VIVO medium (Lonza) with the addition of 1000 IU/mL IL-2 (Bjshsw). All cells were maintained in a humidified incubator at 37°C with 5% CO_2_. Female NOG mice aged 6–8 weeks and weighing 18-20g, were obtained from Zhejiang Vital River Laboratory Animal Technology Co., Ltd. The mice were placed in controlled environments (12-h light/dark cycle; 20-26°C; 40-70% humidity) and had free access to bacteria-free water and food.

A total of 0.1 mL (5×10^7^ cells/mL) of Caski cells were subcutaneously injected into the right flank of mice. When the tumor volume reached approximately 170 mm³, 16 mice (25 mice total) were randomly divided into four groups (n = 4 per group):

Control group: Intratumoral injection of 50 μL PBS every 2 days for a total of 3 doses.

OVV-01 monotherapy group: Intratumoral injection of 50 μL OVV-01 (3×10^6^ PFU per mouse) every 2 days for a total of 3 doses.

TCR-T monotherapy group: Intratumoral injection of 50 μL PBS every 2 days for a total of 3 doses, with a single intravenous injection of 100 μL TCR-T cells (7×10^6^ cells per mouse) on the second day after the first intratumoral administration.

Combination therapy group: Intratumoral injection of 50 μL OVV-01 (3×10^6^ PFU per mouse) every 2 days for a total of 3 doses, with a single intravenous injection of 100 μL TCR-T cells (7×10^6^ cells per mouse) on the second day after the first intratumoral administration.

Tumor volume were measured twice per week for 15 days. At the end of the experiment, animals were euthanized using an overdose of CO_2_.

### Data analysis

The relative normal cell growth inhibition rate was calculated using the formula:

Relative normal cell growth inhibition rate (%) = [(OD_normal cells – OD_tumor cells)/OD_normal cells] × 100%

A bar chart was then generated.

Tumor volume (TV) was calculated using the formula:

TV = 1/2 × a × b², where a and b represent the length and width of the tumor, respectively. Tumor Growth Inhibition (TGITV) was calculated as follows:

TGITV (%) = [1 - (Ti - T0)/(Ci - C0)] × 100%

where:

Ti: Mean tumor volume of the treatment group on day i after drug administration

T0: Mean tumor volume of the treatment group on Day 0 before drug administration

Ci: Mean tumor volume of the control group on day i after drug administration

C0: Mean tumor volume of the control group on Day 0 before drug administration

Cell viability was calculated using the formula:

% of cell surviving = (RLU value of each experimental well/Mean RLU value of the vehicle group) × 100%

Data processing, analysis, and graphical representation were performed using GraphPad Prism 8.0. Statistical comparisons between groups were conducted using the log-rank test, with P < 0.05 considered statistically significant (GraphPad Prism 8, San Diego, CA, USA).

### Ethical approval

Female Balb/c nude mice and Female NOG mice housing and experiments were conducted in accordance with the ethical guidelines formulated by the Animal Experimental Committee of SHANGHAI QI’UP BIOMEDICAL TECHNOLOGY CO.,LTD. Female B-NDG HLA A2.1 mice housing and experiments were conducted in accordance with the ethical guidelines formulated by the Animal Experimental Committee of Biocytogen Pharmaceuticals (Beijing) Co., Ltd.

All animals used in the study were handled in accordance with relevant policies and guidelines for the welfare of laboratory animals.

## Results

### Construction of OVV-01

**Figure 1:**
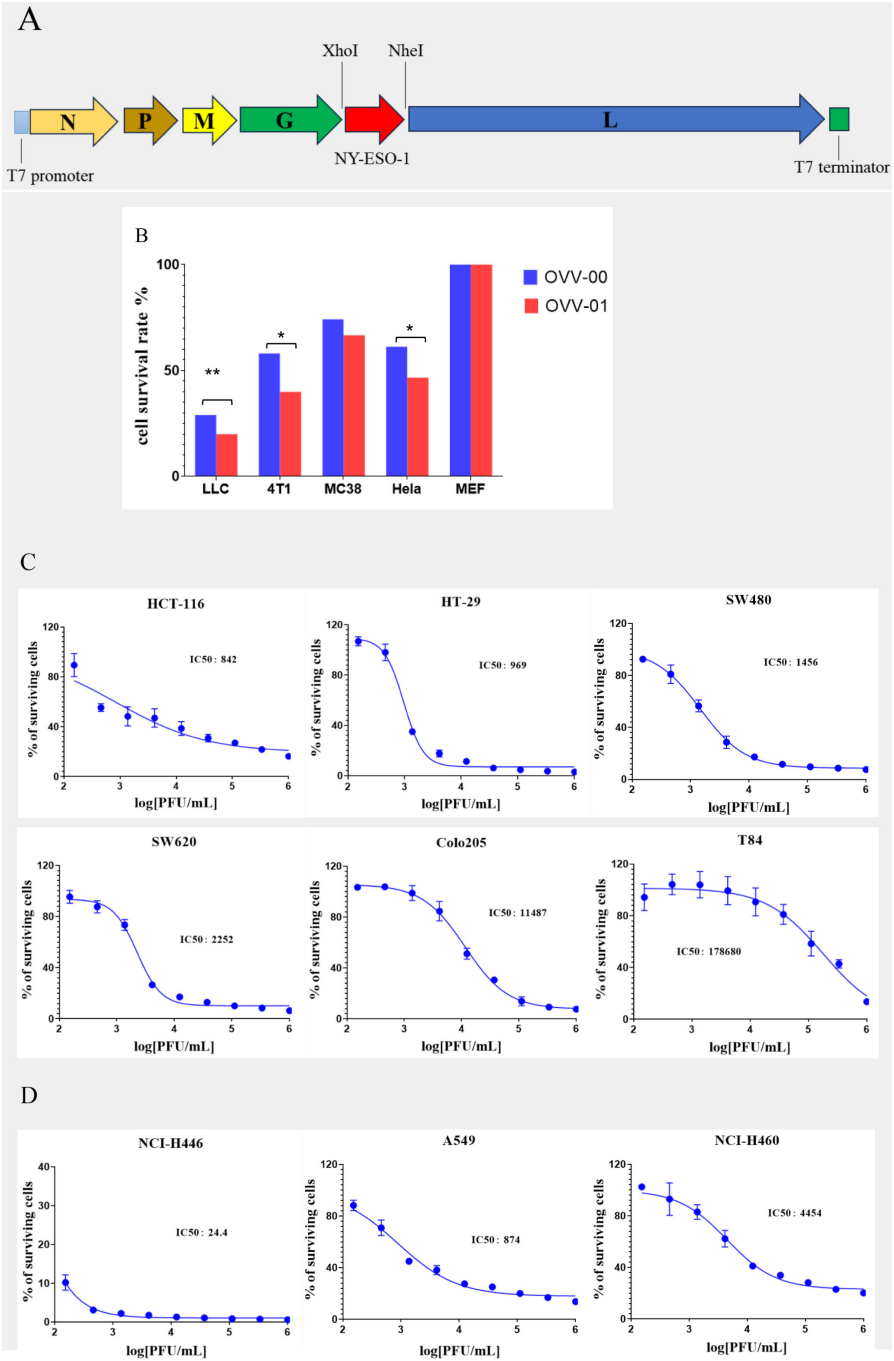
Construction of OVV-01 and tumor cell cytotoxicity. **(A)** pJB-NY-ESO-1 Genome map: the target gene (NY-ESO-1) was integrated into the plasmid at the XhoI and Nhe I enzyme cutting site. **(B)** *In vitro* cytotoxicity results on different cell lines. Cell viability was assessed using the MTT assay after OVV-00 (blue) or OVV-01 (red) of exposure. The bar graph represents the percentage of viable cells relative to the control. *p<0.05 significantly different from OVV-00 (Paired t test), **p<0.01 significantly different from OVV-00 (Paired t test). **(C, D)** Tumor cell survival curves and IC50 values of OVV-01 in human colorectal cell lines **(C)** and human lung cancer cell lines cell lines **(D)**. The cytotoxic effect of OVV-01 on tumor cells was assessed using the CGT reagent to quantify cell viability after 48 hours of OVV-01 treatment. Survival rates were analyzed, and dose-response curves were plotted using GraphPad Prism 8 (San Diego, CA, USA). The half-maximal inhibitory concentration (IC50) was calculated for each cell line.

The backbone plasmid pJB-00 was linearized by double digestion with XhoI and NheI and the NY-ESO-1 gene was inserted between the G protein and L protein genes of the VSV genome (**Figure 1A**). The recombinant virus was generated via cell transfection and subsequently purified through at least two rounds of plaque purification.

### *In vitro* tumor cell cytotoxicity of OVV-01

#### Comparison of the cytotoxicity of OVV-01 and OVV-00 on tumor cells

Multiple cell lines, including MEF (mouse embryonic fibroblasts), LLC (mouse lung cancer cell line), MC-38 (mouse colon cancer cell line), 4T1 (mouse mammary tumor cell line), and Hela (human cervical cancer cell line), were used to evaluate the *in vitro* cytotoxic effects of the vesicular stomatitis virus strains OVV-01 and OVV-00. As shown in **Figure 1B**, *in vitro* cytotoxic assay with a viral infection MOI of 0.1 for 24 hours, both OVV-01 and OVV-00 exhibited minimal toxicity to normal MEF cells. In contrast, OVV-00 inhibited the growth of LLC, MC-38, 4T1, and HeLa tumor cells by 71%, 42%, 26%, and 39%, respectively, whereas OVV-01 inhibited the growth of these cell lines by 80%, 60%, 33%, and 53%, respectively. Overall, the difference between the two viruses was statistically significant (*P* = 0.016). OVV-01 showed a greater inhibitory effect across all tested cell lines than OVV-00, indicating a stronger antitumor activity.

#### Cytotoxic effects of OVV-01 on the colorectal and lung cancer cells

Further studies show that OVV-01 also exhibits infective cytotoxic effects against various other colorectal and lung cancer cell lines. The IC50 values, determined by cell viability assays, are shown in **Table 1**. OVV-01 significantly inhibited the growth of colorectal cancer cells HCT116, HT-29, SW480, SW620, and Colo205, as well as lung cancer cells NCI-H446, A549, NCI-H460, with IC50 values below 1×10^5^ PFU/mL. OVV-01 exhibited slightly weaker infectivity against T84 and HCC827 cells, with an IC50 value of 1.79×10^5^ PFU/mL and 1.07×10^5^ PFU/mL, respectively. NCI-H1975 and NCI-H596 cells showed a higher tolerance to VSV, with their IC50 values exceeding the initial infection concentration of 1×10^6^ PFU/mL. The cell viability curves are shown in **Figures 1C, D**.

**Table 1:** The IC50 values in different tumor cells.

Cancer type	Cell line	Seeding density (cells/well)	Relative IC50 (PFU/mL)
Colon	HCT-116	10000	842
	HT-29	10000	969
	SW480	10000	1456
	SW620	10000	2252
	Colo205	10000	11487
	T84	10000	178680
Lung	NCI-H446	10000	24
	A549	10000	874
	NCI-H460	10000	4454
	HCC827	10000	107026
	NCI-H1975	10000	2271813
	NCI-H596	10000	12291527

### Specificity of OVV-01 replication in tumors

**Figure 2:**
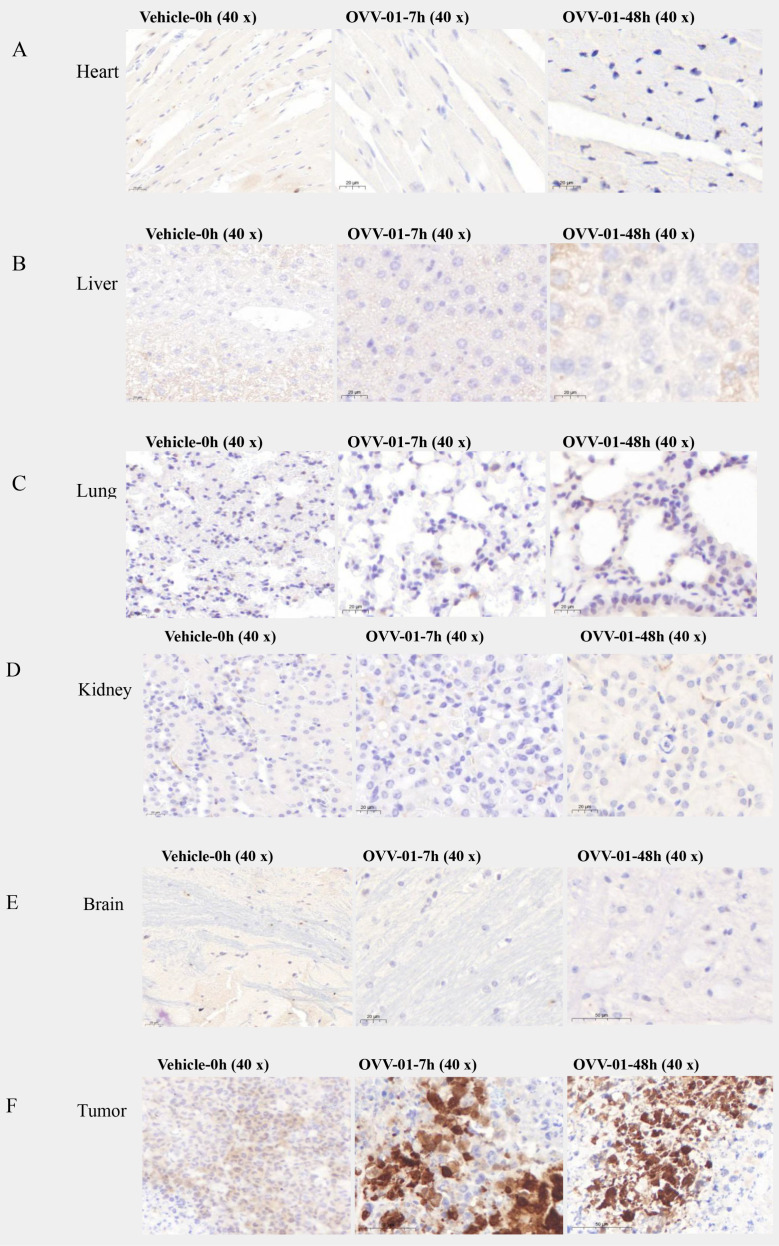
Summary of immunohistochemical findings. **(A–E)** Representative immunohistochemical profiles of normal tissues (heart, liver, lung, kidney, brain) showing no detectable immunoreactivity at baseline (vehicle group, 0 h) or post-treatment time points (7 h and 48 h). **(F)** Tumor immunoreactivity dynamics over time. At baseline (vehicle, 0 h), weak cytoplasmic staining was detected (1+, 15%). At 7 h post-treatment, staining intensity increased (3+, 30%). At 48 h, strong staining persisted (3+), but the percentage of positive cells decreased (8%).

To investigate the biodistribution of OVV-01 after its administration, we established H22 syngeneic tumor model with a single intravenous dose. H22 cells were subcutaneously injected into the right flank of mice at a density of 2×10^5^ cells/0.1 mL/mouse. When the tumor volume reached approximately 200 mm³, the mice were randomly grouped, and a single intravenous injection of OVV-01 at a dose of 3×10^8^ PFU/mouse was administered on the same day. Tissue samples were collected at 7 hours and 48 hours post-administration for immunohistochemical analysis to detect NY-ESO-1 expression. The immunohistochemical results showed varying degrees of positive NY-ESO-1 protein expression in tumor tissues, while no expression was observed in normal tissues such as the heart, liver, spleen, lung, kidney, brain, and uterus, as shown in **Figure 2**. In the vehicle-0h control group, NY-ESO-1 immunostaining was undetectable in normal tissues and organs, whereas the plasma of tumor cells displayed weak positivity (1+ intensity, 15% positivity rate). Strikingly, the OVV-01-7h treatment group exhibited complete absence of NY-ESO-1 in normal compartments but showed a marked upregulation of NY-ESO-1 expression in the plasma of tumor cells, with strong immunoreactivity (3+ intensity) observed in 30% of tumor cells compared to the vehicle control. Notably, while the OVV-01-48h group similarly lacked NY-ESO-1 staining in normal tissues, tumor-specific expression persisted at a reduced positivity rate (8%) despite sustained high intensity (3+), indicating a potential time-dependent attenuation of NY-ESO-1 expression. Critically, these results collectively demonstrate that VSV selectively replicates in tumor tissues while sparing normal tissues, highlighting its tumor-targeting specificity and therapeutic potential.

### OVV-01 potentiates antitumor immune responses through dual mechanisms: boosting tumor-infiltrating lymphocyte recruitment and enhancing peripheral blood T cell activation

To better determine the effects of OVV-01 on tumor-infiltrating lymphocytes (TILs) and anti-tumor immune responses, we established a humanized immune system mouse tumor model. B-NDG HLA A2.1 mice were intravenously injected with human PBMCs, followed by subcutaneous injection of colon cancer HCT116 cells (5×10^6^ cells/0.1 mL/mouse) into the right flank. When the average tumor volume reached approximately 144 mm³, the mice were randomly grouped and OVV-01 treatment began on the same day. The treatment was administered every two days for a total of three doses. Peripheral blood and tumor tissues were collected on day 7, 14, and 21 post-treatment to assess T cell number and activation state.

**Figure 3:**
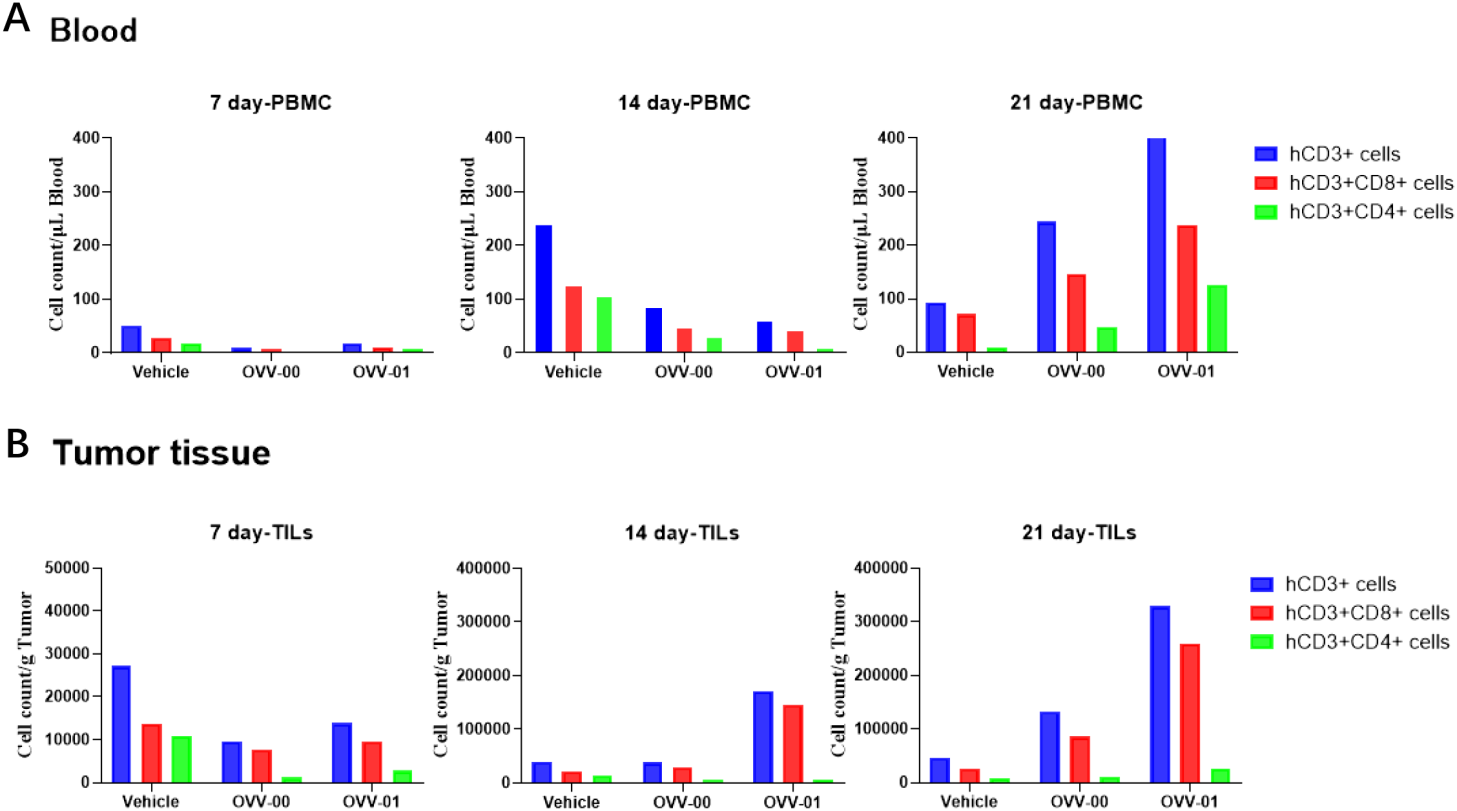
Flow cytometric analysis of T cell subtypes in mouse peripheral blood **(A)** and tumor tissues **(B)**. Quantitative analysis of T cell populations, including hCD3+ T cells, hCD3+CD8+ T cells, and hCD3+CD4+ T cells, was performed at 7, 14, and 21 days post-treatment. Data are presented as mean ± standard error of the mean (n = 6).

The flow cytometry results of peripheral blood and TILs in tumor tissues are shown in **Figures 3A, B**. After treatment, on day 7, 14, and 21, both the OVV-00 and OVV-01 treatment groups showed an increase in hCD3+ T cells, hCD3+CD8+ T cells, and hCD3+CD4+ T cells in peripheral blood and tumor TILs over time. For the TILs on day 14, the OVV-01 treatment stimulated significantly higher levels of hCD3+ T cells and hCD3+CD8+ T cells compared to the control and OVV-00 groups. By day 21, the OVV-01 group exhibited markedly higher levels of hCD3+ T cells, hCD3+CD8+ T cells, and hCD3+CD4+ T cells in both peripheral blood and tumor compared to the control and OVV-00 groups, indicating that OVV-01 has a stronger immunostimulatory effect than OVV-00. Additionally, at the 14-day time point, PBMC analysis revealed elevated levels of hCD3+ T cells and hCD3+CD8+ T cells in both the control and treatment groups, with higher levels observed in the control group compared to the treatment group. However, in TILs, the OVV-01 treatment group exhibited significantly higher levels of hCD3+ T cells and hCD3+CD8+ T cells compared to both the control group and the OVV-00 treatment group. These results suggest enhanced infiltration of T cells into tumor tissues following OVV-01 administration.

We also performed flow cytometry to detect NY-ESO-1 TCR-T cells in both the peripheral blood and tumor tissues of mice. We observed that 1/3 of the mice showed detectable NY-ESO-1 TCR-T cells on both day 7 and day 21 post-treatment (results not shown here), suggesting that OVV-01 can induce the generation of NY-ESO-1-specific T cells in tumor tissues.

### Anti-tumor effects of OVV-01 combined with NY-ESO-1 TCR-T cells

We used a co-culture model of TCR-T cells with OVV-01-infected tumor cells to study the combined tumor-killing effects of OVV-01 and NY-ESO-1 TCR-T cells. The antigen epitope targeted by NY-ESO-1 TCR-T is HLA-A*02-restricted. In this study, we selected the HLA-A*02-positive cell lines A673 and K562 as target cell lines for TCR-T cells.

#### OVV-01 induces overexpression of NY-ESO-1 protein on tumor cell surfaces

**Figure 4:**
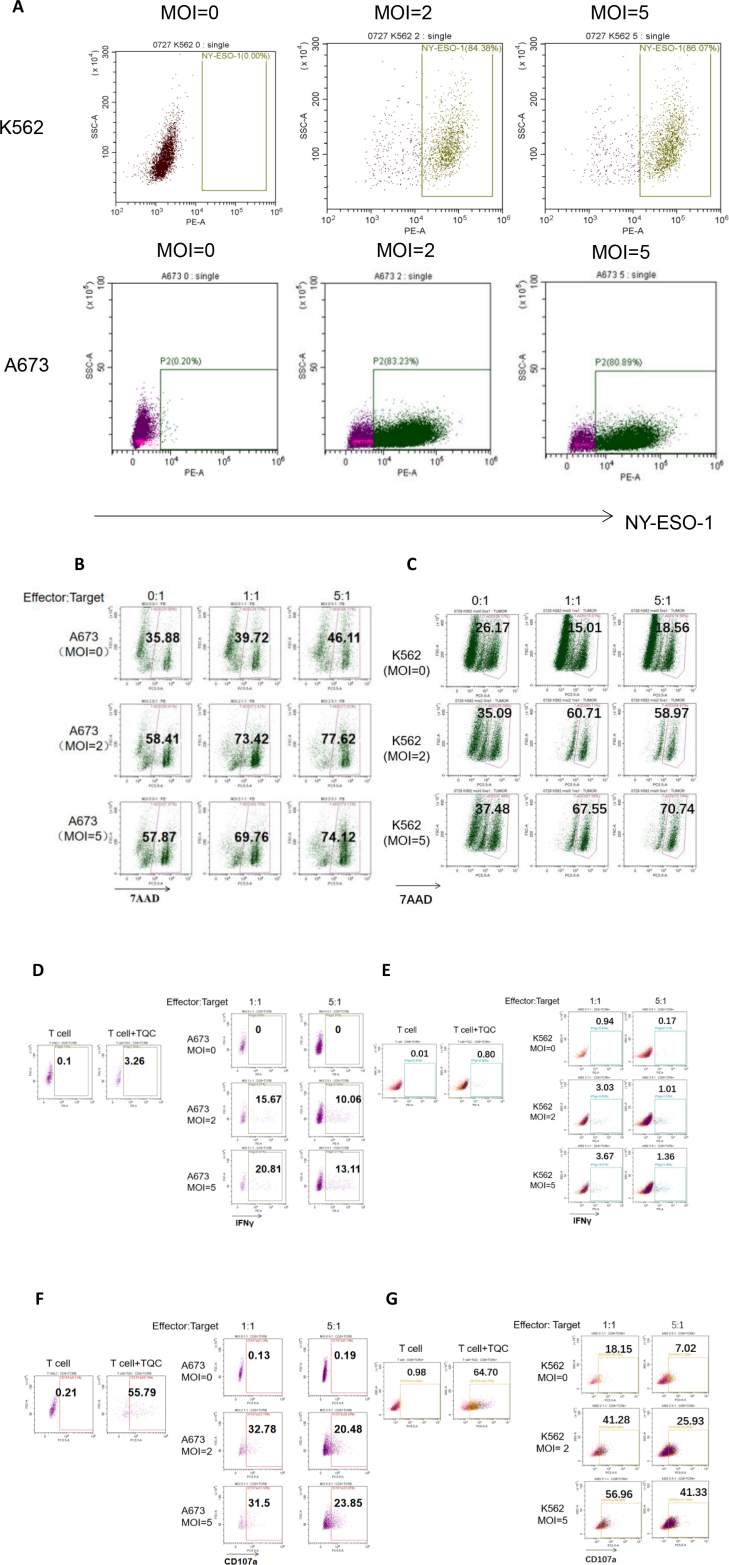
Flow cytometric analysis of the Cytotoxic Effects of OVV-01 in Combination with TCR-T Cells on Tumor Cells. **(A)** Expression of NY-ESO-1 in OVV-01 (MOI=0, 2 and 5) infected K562 cells (top) for 48h and A673 cells (bottom) for 24 h. **(B, C)** Cytotoxic activity of TCR-T cells against OVV-01-infected tumor cells was quantified using flow cytometric analysis. A673 **(B)** and K562 **(C)** cells were infected with OVV-01 (MOI = 0, 2, 5) for 24 hours. After 48 hours of co-culture at an effector-to-target (E:T) ratio of [1:1 or 5:1], cells were harvested and stained with 7-AAD to discriminate dead populations. **(D–G)** OVV-01 infection enhances IFNγ **(D, E)** secretion and CD107a **(F, G)** expression by TCR-T cells in a short-term co-culture assay. For positive control, T cell activation was performed using CD3/28 beads combined with specific TCR-T cell antigenic peptide stimulation (T Cell Activation Cocktail, TQC). A673 and K562 cells were infected with OVV-01 (MOI = 0, 2, 5) for 24 hours. Then TCR-T cells were co-cultured with infected cells at an effector-to-target (E:T) ratio of [1:1 or 5:1] for 5 hours. Flow cytometry was used to analyze the expression of IFN-γ and CD107a in cells.

We used flow cytometry to detect the expression of NY-ESO-1 on K562 and A673 cells in the co-culture system. As shown in **Figure 4A**, the results revealed that after 48 hours of OVV-01 infection, approximately 85% of K562 cells were positive for NY-ESO-1, while after 24 hours of OVV-01 infection, about 80% of A673 cells were positive for NY-ESO-1. These findings demonstrate that OVV-01 can effectively mediate the overexpression of NY-ESO-1 in target cell lines, leading to the presentation of NY-ESO-1 antigen peptides that can be recognized by TCR-T, thereby activating TCR-T cells to kill target cells.

#### Synergistic effect of OVV-01 on tumor killing by NY-ESO-1 TCR-T cells

Killing of tumor cells after 48 hours of co-treatment with OVV-01 and TCR-T cells was assessed using flow cytometry. As shown in **Figures 4B, C**, the results revealed that in A673 target cells, approximately 35% was 7AAD positive without oncolytic virus infection. The oncolytic virus infection (MOI=2 and 5, E:T =0) increased the percentage to around 58%. When co-cultured with TCR-T cells (Effector) (E:T =1 and 5 compared to E:T =0), the percentage increased to 69-77%. In K562 target cells, approximately 26% was 7AAD positive without oncolytic virus infection. OVV-01 alone (MOI=2 and 5, E:T =0) increased the percentage to around 35%. When co-cultured with TCR-T cells (MOI=2 and 5, E:T =1 and 5), the percentage further increased to around 60-70%. Therefore the combination of oncolytic virus and T cells exhibits an additive cytotoxic effect on tumor cells, with increasing MOI and E:T ratios both contributing to enhanced tumor cell killing.

IFNγ is a crucial cytokine released by effector T cells upon activation. OVV-01 mediated the overexpression of NY-ESO-1 in target cells. If the antigen peptides recognized by TCR-T cells are correctly presented by HLA molecules on the surface of target cells, TCR-T cells can be activated to secrete IFNγ. As shown in **Figures 4D, E**, under co-culture conditions, after OVV-01 infection of A673 and K562 cells (MOI=2 and MOI=5), the secretion of IFNγ by TCR-T cells was significantly increased (21%, 3.67%, respectively) compared to the control group (uninfected). Additionally, A673 cells stimulated a higher IFNγ secretion than K562 cells, consistent with the stronger killing of A673 cells by TCR-T cells observed in **Figures 4D, E**. Furthermore, at an effector-to-target cell ratio (E:T) of 1, TCR-T cells secreted higher levels of IFNγ compared to an E:T ratio of 5 (20.81% vs 13.11% for A673, 3.67% vs 1.37 for K562). Together, these results indicate that endogenous overexpression of NY-ESO-1 significantly influences (21% vs 0 for A673, 3.67% vs 0.94% for K562) the activation of TCR-T cells and a relatively lower numbers of effector T cells can lead to better activation.

Upon activation, CD8+ effector T cells undergo degranulation to release perforin and granzymes to kill target cells. Effector T cells in a degranulated state were stained by CD107a antibody followed by flow cytometry. As shown in **Figures 4F, G**, as a positive control, direct stimulation of TCR-T cells with the corresponding antigen peptide increased CD107a expression in TCR-T cells. Under co-culture conditions, after oncolytic virus infection of A673 and K562 cells (MOI=2 and MOI=5), the CD107a positivity rate of TCR-T cells was significantly increased (31.5% vs 0.13 for A 673, 56.96% vs 18.15% for K562) compared to the control group (without OVV-01 infection). Additionally, consistent with previous findings, the CD107a positivity rate was higher at an E:T ratio of 1 compared to an E:T ratio of 5. Furthermore, differences in CD107a positivity were observed between A673 and K562 cells, with K562 showing higher CD107a expression across all groups.

In summary, these results show that OVV-01 can successfully mediate the expression of NY-ESO-1 in tumor target cell lines. The NY-ESO-1 expressing tumor cells are recognized and killed by effector T cells carrying TCR specific for NY-ESO-1 peptide presented by HLA-A*02:01.

### *In vivo* efficacy evaluation of OVV-01 in tumor-bearing mice

#### OVV-01 inhibits tumor growth in hepatocellular carcinoma-bearing mice.

**Figure 5:**
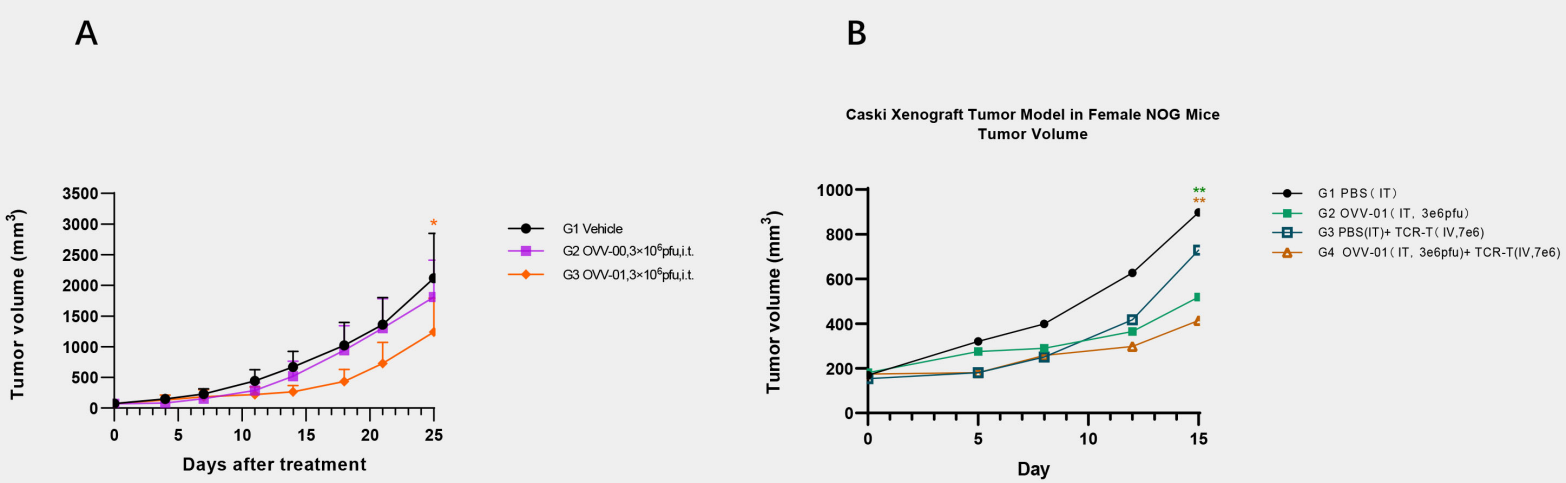
Tumor growth profiles. **(A)** Tumor Volume Growth Curve of Murine Hepatocellular Carcinoma (H22) Xenografts. Data are expressed as mean ± SD(n=6). * p<0.05 significantly different from OVV-00 (Paired t test). **(B)** Changes in animal tumor volume in Caski (HLA-A2+/NY-ESO-1-) human cervical cancer model. Data are expressed as mean ± SD (n = 4). **p<0.01 significantly different from G1 (Bonferroni’s multiple comparisons test).

We established a murine hepatocellular carcinoma H22 syngeneic tumor model to evaluate the *in vivo* antitumor activity of vesicular stomatitis virus OVV-01 and OVV-00. H22 cells (2×10^5^ cells/0.1 mL/mouse) were subcutaneously injected into the right flank of mice. When the tumor volume reached approximately 60 mm³, the mice were randomly assigned to different groups, and treatment was initiated on the same day. The OVVs were administered every two days for a total of seven doses. Tumor volume change curves are shown in **Figure 5A**. At the study endpoint (Day 25), the tumor growth inhibition rate (TGITV %) in the OVV-00 treatment group (3×10^6^ PFU/mouse) was 14.88%, demonstrating a modest inhibitory effect on H22 tumor growth in mice. In contrast, the TGITV % in the OVV-01 treatment group (3×10^6^ PFU/mouse) was 42.92%, showing a significant higher tumor suppression (P = 0.037). These results indicate that at a dosage of 3×10^6^ PFU/mouse, OVV-01 is more effective than OVV-00, suggesting that tumor antigen expression may enhance the antitumor efficacy of recombinant VSV.

#### OVV-01 combined with NY-ESO-1 TCR-T cells inhibits tumor growth in tumor-bearing mice

To investigate the *in vivo* efficacy of the combined treatment of OVV-01 and NY-ESO-1 TCR-T cells, we established a Caski (HLA-A2+/NY-ESO-1-) human cervical cancer cell line NOG mouse subcutaneous xenograft model. Caski cells were subcutaneously injected into the right flank of mice at a density of 5×10^7^ cells/0.1 mL/mouse. When the average tumor volume reached approximately 169 mm³, the mice were randomly grouped, and treatment began on the same day. The control group received intratumoral injections of PBS, while the OVV-01 monotherapy group received intratumoral injections of 3×10^6^ pfu/mouse, every 2 days for a total of 3 doses. The TCR-T monotherapy group received intratumoral injections of PBS every 2 days for 3 doses, with a single intravenous injection of TCR-T cells at 7×10^6^ cells/mouse on day 2 of treatment. The combination therapy group received intratumoral injections of OVV-01 at 3×10^6^ pfu/mouse every 2 days for 3 doses, along with a single intravenous injection of TCR-T cells at 7×10^6^ cells/mouse on day 2 of treatment. Tumor growth was monitored, and on day 15 post-treatment, the tumor growth inhibition rates for the TCR-T monotherapy, OVV-01 monotherapy, and OVV-01 + TCR-T combination therapy groups were 21.17%、53.66% and 67.18%, respectively. The tumor volume change curves are shown in **Figure 5B**. Both OVV-01 monotherapy and the combination therapy significantly inhibited tumor growth, with the combination group demonstrating a notable additive effect.

## Discussion

Our research demonstrates that the recombinant VSV virus expressing the NY-ESO-1 antigen gene exhibits selective replication in tumor tissues, with minimal impact on normal cells or tissues. It effectively kills tumor cells and significantly inhibits tumor growth in tumor-bearing mice. Additionally, this virus exhibits potent immunostimulatory effects, promoting the proliferation of CD3+, CD8+, and CD4+ T cells. This study also explores the potential of combining OVV-01 with TCR-T cell therapy. The results show that OVV-01 successfully mediates the expression of NY-ESO-1 in tumor target cell lines. The overexpressed NY-ESO-1 protein is presumably processed into antigenic peptides and presented on the cell surface by HLA class I molecules, enabling recognition and killing by effector T cells carrying the corresponding TCR. The combined application of OVV-01 and TCR-T cells demonstrates a clear additive effect in killing tumor cells both *in vitro* and *in vivo*. In tumor-bearing mouse models, the combination therapy significantly inhibits tumor growth compared to TCR-T cell therapy alone.

The safety of the VSV backbone for clinical use has been extensively studied (https://classic.clinicaltrials.gov). NY-ESO-1 is a tumor-associated antigen (TAA) and expressed in approximately one-quarter to one-third of lung cancers, melanomas, ovarian cancers, esophageal cancers, bladder cancers, and prostate cancers, and its expression is associated with high-grade tumors. NY-ESO-1 is a highly immunogenic antigen capable of eliciting strong immune responses. Leveraging the selective replication of VSV in tumor cells and expression of NY-ESO-1 in tumor cells, combining NY-ESO-1 expression with NY-ESO-1 TCR-T cell therapy has the potential to activate specific immune responses and overcome the limitations of current NY-ESO-1-targeted therapies due to heterogeneous antigen expression, thereby offering potential therapeutic benefits for such cancer patients.

The application of VSV as an oncolytic virus in cancer therapy holds great promise, but challenges remain, such as the development of more convenient delivery methods, optimizing dosing frequency, dosage, and the generation of neutralizing antibodies. Future research could focus on dose optimization and adjusting administration strategies to more precisely balance efficacy and toxicity, exploring personalized treatment regimens to maximize patient benefits. In terms of antigen engineering, VSV can be designed to express various tumor-associated antigens, not limited to NY-ESO-1, thereby expanding its range of indications to cover more types of solid tumors and hematologic malignancies. Combining VSV with immune checkpoint inhibitors (such as anti-PD-1/PD-L1 or anti-CTLA-4 antibodies) may further alleviate immune suppression in the tumor microenvironment and enhance the activity of effector T cells. Combining VSV with CAR-T or TCR-T cell therapies could generate powerful anti-tumor activities through direct tumor killing by VSV, local antigen expression and systemic immune activation (39, 40). Additionally, VSV can be further optimized by introducing specific cytokines (such as IL-12 or GM-CSF) or immune-modulating molecules to enhance its immunostimulatory activity both within and outside the tumor. By delving deeper into the mechanisms of immune evasion and tolerance of oncolytic viruses, improvements in viral design can enhance its selectivity and persistence. Exploring strategies that combine oncolytic viruses with immune checkpoint inhibitors and adoptive cell therapies could strengthen anti-tumor immune responses and improve the durability and safety of treatments. Such multimodal therapeutic approaches are expected to become a key direction in future cancer immunotherapy (35, 41).

In addition to our preclinical findings, it is noteworthy that OVV-01 has already entered investigator-initiated trials (IITs) at multiple hospitals. The preliminary clinical outcomes, primarily focusing on safety and feasibility, were recently reported by Hua et al. in *Journal for Immunotherapy of Cancer* (2023, Vol. 13, Issue 6). Their study confirmed that OVV-01 was well tolerated in patients with advanced solid tumors, establishing a favorable safety profile for subsequent translational development.

Building upon this clinical safety foundation, our current study provides mechanistic and efficacy-focused insights into OVV-01. Specifically, we demonstrate its tumor-selective infectivity, NY-ESO-1 transgene expression, and capacity to enhance antigen-specific T-cell responses *in vitro* and *in vivo*. Furthermore, we explore a novel therapeutic approach by combining OVV-01 with NY-ESO-1-specific TCR-T cell therapy, which achieved synergistic antitumor effects across multiple tumor models. Collectively, these findings not only complement the existing clinical data but also support the rational design of combination strategies for future clinical development.

Nevertheless, several limitations of this study should be acknowledged. Although Caski cells are HLA-A2-positive *in vitro*, we did not re-evaluate HLA-A2 expression in tumors after engraftment *in vivo*. Considering that tumor cells may downregulate MHC class I molecules during tumor progression (32, 42), the absence of HLA-A2 confirmation at the treatment stage represents a potential limitation, as it may affect the efficiency of NY-ESO-1 TCR-T cell recognition. Additionally, we did not assess the proliferation, persistence, or activation status of transferred TCR-T cells *in vivo*, which would have provided mechanistic insight into T cell functional engagement following treatment. In future studies, we plan to incorporate dynamic monitoring of target antigen expression and comprehensive characterization of TCR-T cell responses in the tumor microenvironment, to better elucidate the mechanism of action and optimize this combination strategy.authorship contribution statement

## Data Availability

The raw data supporting the conclusions of this article will be made available by the authors, without undue reservation.
